# Bronchial Obstruction in Immunoglobulin G4-Related Disease

**DOI:** 10.1016/j.atssr.2024.11.011

**Published:** 2024-12-11

**Authors:** Takaya Sato, Kentaro Minegishi, Naota Okabe, Fumie Osuga, Keigo Sudo, Masaya Sogabe, Shunsuke Endo, Hiroyoshi Tsubochi

**Affiliations:** 1 General Thoracic Surgery, Jichi Medical University Saitama Medical Center, Saitama, Japan; 2 Pathology, Jichi Medical University Saitama Medical Center, Saitama, Japan

## Abstract

Immunoglobulin G4-related disease (IgG4-RD) is a recently identified systemic fibroinflammatory disorder affecting various organs throughout the body. IgG4-related lung disease is a relatively common manifestation in IgG4-RD and presents with 4 primary pulmonary patterns: nodular, ground-glass opacities, interstitial disease, and peribronchovascular thickening. Peribronchovascular thickening is a frequent pattern in thoracic IgG4-RD involvements. This case report describes bronchial obstruction as a consequence of IgG4-RD in a 65-year-old man with no personal or family history of autoimmune disease. Thoracoscopic segmentectomy was performed, which led to the diagnosis of IgG4-RD.

Immunoglobulin Ig4-related disease (IgG4-RD) is a recently characterized systemic fibroinflammatory disorder affecting various organs throughout the body. It was originally described in 1951 as chronic autoimmune pancreatitis.^1^ Recently, IgG4-RD has been recognized and was formally defined by classification criteria in 2019.^2^ Pulmonary involvement is relatively common in IgG4-RD. This report presents a case of bronchial obstruction caused by IgG4-RD in a 65-year-old man diagnosed by thoracoscopic lung segmentectomy. This research adhered to the ethical standards and guidelines set by Jichi Medical University Saitama Medical Center and received approval from the Institutional Review Board (S24-060).

The patient was a 65-year-old man who presented to our hospital with an incidental finding of an abnormal shadow in the left lower lung field. His medical history included type 2 diabetes mellitus and hypertension. He had a smoking history of 20 cigarettes per day for 20 years. Computed tomography showed the obstruction of the left B8 bronchial entrance and peripheral atelectasis (Figure 1). Bronchoscopy revealed obstruction in the B8 bronchus and stenosis in the B9 and 10 bronchi (Figure 2). Because a tumor-related obstruction could not be excluded, a thoracoscopic left basal segmentectomy was performed for diagnostic and therapeutic purposes. Intraoperative findings revealed fibrous thickening of the peribronchovascular bundle around the basal bronchus.

**Figure 1:**
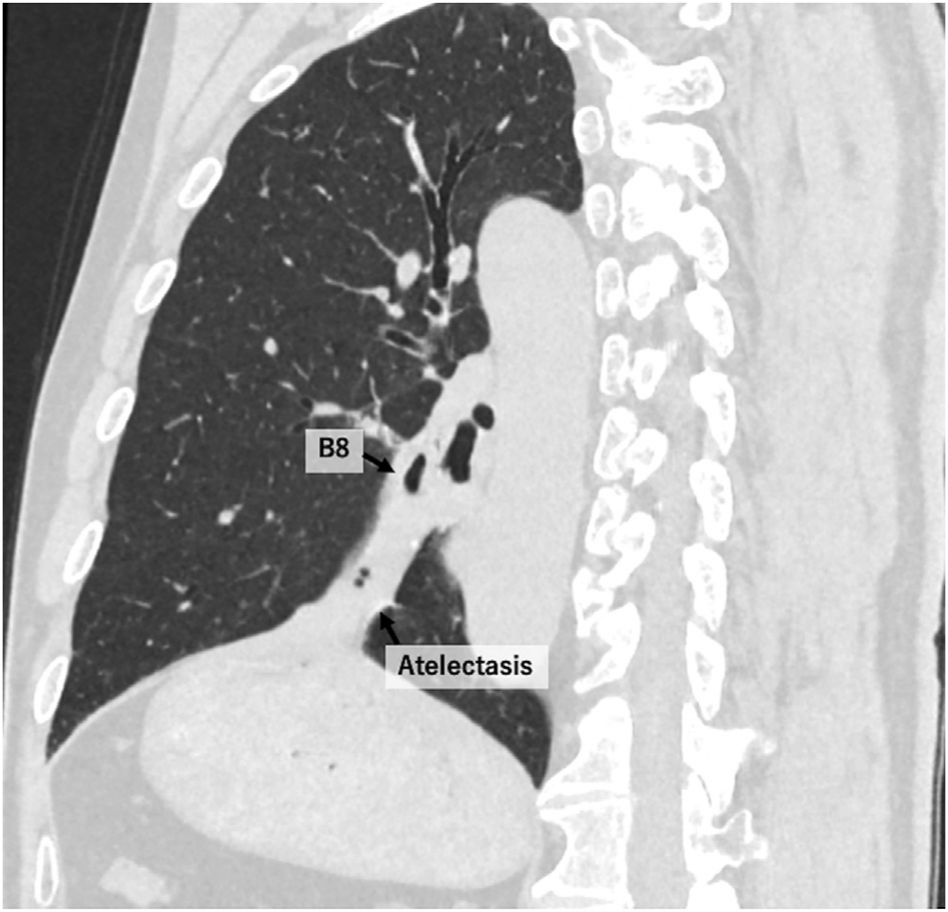
Chest computed tomography showed B8 bronchial disruption with associated peripheral atelectasis.

**Figure 2:**
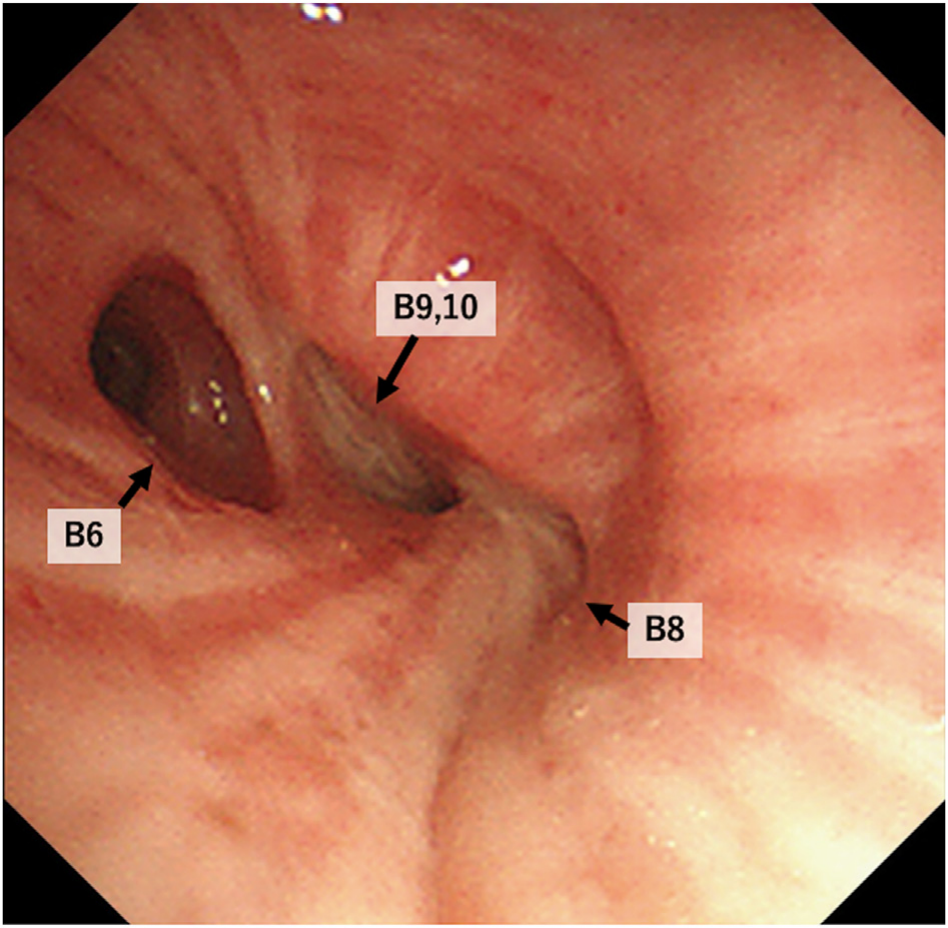
Bronchoscopy showed obstruction of B8 and intraluminal stenosis of B9-10. The bronchial epithelium was preserved.

The operative time was 123 minutes. Blood loss was 30 mL. Pathologic analysis showed lymphoplasmacytic infiltration with >200 IgG4-positive cells per high-power field, and the IgG4-to-IgG ratio was ≥0.4 (Figure 3). Postoperative blood tests showed that serum IgG4 was high at 512 mg/dL. Based on these pathologic and biological results, he was diagnosed with bronchial obstruction caused by IgG4-RD.

**Figure 3:**
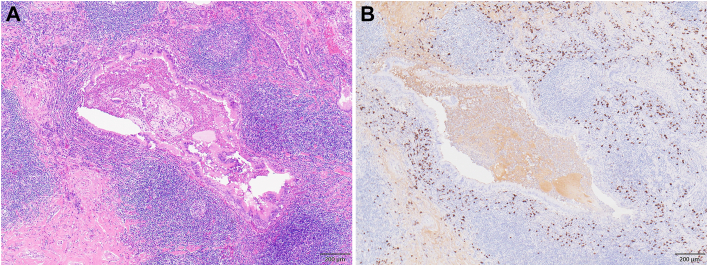
Pathologic findings. (A) Hematoxylin and eosin staining showed bronchial compression due to plasma cell infiltration and phlegm retention within the bronchiole. (B) Immunoglobulin G4 (IgG4) immunohistochemical staining revealed significant infiltration of IgG4-positive plasma cells, with >200 IgG4-positive cells per high power field and an IgG4/immunoglobulin G ratio >40%. Scale bar: 200 μm.

The postoperative course was uneventful, and he was discharged on postoperative day 9. A comprehensive evaluation found no additional abnormalities related to IgG4-RD. In the 4 months since the surgery, no adverse events have been observed.

## Comment

IgG4-RD is an immune-mediated fibroinflammatory disease characterized by organ enlargement and elevated serum IgG4 levels. It is a multiorgan disease that mimics malignant, infectious, and inflammatory disorders. Almost any organ or tissue can be affected, including the pancreas, hepatobiliary system, salivary and lacrimal glands, kidney, and retroperitoneum.

The first cases of IgG4-RD were described in 1961 when Sarles and colleagues^1^ reported a chronic autoimmune pancreatitis characterized by fibrosis and lymphoplasmacytic infiltration. In 2006, Kamisawa and Okamoto^2^ reported the presence of an IgG4-related sclerosing disease characterized by fibroinflammatory lesions in both pancreatic and extrapancreatic tissues that are rich in IgG4. Currently, the term IgG4-RD is widely accepted and is defined according to specific classification criteria published in 2019.^3^

Although IgG4-RD can affect various organs, thoracic involvement is relatively common, affecting an estimated 30% of patients with systemic IgG4-RD.^4^ Dai and colleagues^5^ proposed 7 primary patterns of IgG4-related lung disease, which can coexist. Four of these patterns involve the lungs: nodular, ground-glass opacities (GGO), interstitial disease, and peribronchovascular thickening. The remaining 3 patterns involve extrapulmonary thoracic structures: lymph nodes, pleura, and retromediastinum.^5^ The bronchovascular type is characterized by the thickening of the bronchovascular bundle and interlobular septa, as in this case.

Treatment is recommended for all patients with symptomatic IgG4-related lung disease. Localized forms of the disease, such as pulmonary nodules or inflammatory pseudotumors, can often be effectively treated with surgical excision. In other cases, the treatment of thoracic IgG4-RD disease typically involves immunosuppressive therapy. However, it has been reported that approximately half of patients treated with immunosuppressive agents experience no improvement or relapse after treatment is discontinued.^6^

In addition to tumors and infections, the differential diagnosis for bronchial obstruction includes several autoimmune diseases, including relapsing polychondritis, sarcoidosis, and granulomatosis with polyangiitis.^7^ Here we report bronchial obstruction due to peribronchovascular thickening of IgG4-RD.

In this patient, the lesion was localized, and the possibility of neoplastic change could not be ruled out, so surgery was performed to find and treat the cause. Further accumulation of cases of bronchial obstruction due to IG4-RD is necessary in the future, because changes such as those seen in this case may occur. It is also important to include IgG4-RD in the differential diagnosis of bronchial obstruction, and a comprehensive systemic evaluation must be performed before determining a treatment plan.

## References

[1] Sarles H., Sarles J.C., Muratore R. (1961). Chronic inflammatory sclerosis of the pancreas—an autonomous pancreatic disease?. Am J Dig Dis.

[2] Kamisawa T., Okamoto A. (2006). Autoimmune pancreatitis: proposal of IgG4-related sclerosing disease. J Gastroenterol.

[3] Wallace Z.S., Naden R.P., Chari S. (2020). The 2019 American College of Rheumatology/European league against rheumatism classification criteria for IgG4-related disease. Arthritis Rheumatol.

[4] Ryu J.H., Sekiguchi H., Yi E.S. (2012). Pulmonary manifestations of immunoglobulin G4-related sclerosing disease. Eur Respir J.

[5] Dai I., Yoh Z., Hitoshi A. (2009). Immunoglobulin G4-related lung disease: CT findings with pathologic correlations. Radiology.

[6] Jia L., Yuxiang L., Ximing S. (2021). Clinicopathological characteristic of IgG4-related disease. BMC Pulm Med.

[7] Hattab Y., Perez de Salmeron S., Mal E. (2024). Benign airway obstruction: a clinical practice review of causes and managements principles. AME Med J.

